# Arfs on the Golgi: four conductors, one orchestra

**DOI:** 10.3389/fmolb.2025.1612531

**Published:** 2025-07-31

**Authors:** Selma Yilmaz Dejgaard, John F. Presley

**Affiliations:** ^1^ Department of Anatomy and Cell Biology, McGill University, Montreal, QC, Canada; ^2^ Department of Medical Biology, Near East University, Nicosia, Cyprus

**Keywords:** ARF, golgi, secretory pathway, COPI, redundancy, ARF1, ARF4, ARF3

## Abstract

Arfs are small Ras-superfamily proteins important for regulating membrane trafficking including the recruitment of vesicular coats as well as a diverse range of other functions. There are five Arfs in humans: two Class I Arfs (Arf1 and Arf3), two Class II Arfs (Arf4 and Arf5) and one Class III Arf (Arf6), with Class I and Class II Arfs present on the Golgi apparatus among other locations. These Golgi Arfs (Arf1, Arf3, Arf4 and Arf5) are highly similar in sequence, and knockout studies have established a complex pattern of redundancy, with Arf4 alone able to support cell survival in tissue culture. Moreover, adding to the complexity, functions of Arfs on distinct membranes can involve non-overlapping sets of effectors (e.g., COPI on *cis*-Golgi membranes and clathrin adaptors on *trans*-Golgi network). The three classes of Arfs are found in most metazoans, suggesting biologically important specialization the details of which are beginning to emerge. This review examines recent studies using siRNA and CRISPR/Cas9 knockouts of mammalian Arfs combined with functional assays of the secretory pathway in the context of detailed localization of fluorescently-tagged Arfs by fluorescent and super-resolution microscopy and the existing literature using more conventional techniques. We suggest that specificity of effector recruitment involves additional membrane determinants which need to be considered in future studies.

## Introduction

Ras-superfamily GTPases, particularly Rab (Zerial and McBride, 2001) and Arf/Arl (Kahn et al., 2006) family proteins play an important role in intracellular trafficking. Rab-family proteins play a variety of roles, including recruitment of motor proteins, tethering factors and SNARE proteins involved in vesicle movement, and recognition and fusion between membranes (Zerial and McBride, 2001). Arf/Arl proteins also play a variety of roles. The first identified Arf, Arf1, was first identified as a factor required for ADP-ribosylation of adenylate cyclase (Schleifer et al., 1982; Kahn and Gilman, 1984) subsequently determined to be a small GTP-binding protein (Kahn and Gilman, 1986). The physiological function of Arf1 was unclear until subsequent work established its importance in recruitment of coat proteins involved in vesicle budding (Helms and Rothman, 1992; Donaldson et al., 1991; Teal et al., 1994).

In general, Ras-superfamily GTPases are small lipid-anchored proteins that act as molecular switches, with most cycling between GTP-bound active states and GDP-bound inactive states (Bourne, 1995; Tetlow et al., 2013). The Ras family protein is often retained on membrane by a lipid anchor during at least the GTP-bound portion of its cycle (Tetlow et al., 2013). A GTPase exchange factor (GEF) is typically required to activate it causing it to release GDP and bind GTP, rendering the protein active (Bourne, 1995). While in the active form, it can bind effectors through either a switch 1 or a switch 2 region (Polakis and McCormick, 1993), allowing the GTPase to mediate its action (Bourne, 1995). In the case of Arfs, effectors can include a diverse range of proteins, such as COPI (Helms and Rothman, 1992; Donaldson et al., 1991; Teal et al., 1994; Serafini et al., 1991) or clathrin coats (Stamnes et al., 1993; Traub et al., 1993), lipid-modifying enzymes (Ktistakis et al., 1995), spectrins (De Matteis and Morrow, 2000; Godi et al., 1998), and tethering proteins (Gillingham et al., 2004; Short et al., 2005). When membrane-associated, Arfs and Arf/Arl family proteins are typically anchored in the membrane by a N-terminal myristylation (Donaldson and Jackson, 2011) in contrast to other Ras-superfamily proteins, which are typically membrane-anchored via C-terminally attached fatty acid chains (Tetlow et al., 2013). Most Ras-superfamily GTPases, Arfs included, possess no or very weak intrinsic GTPase activity (Antonny et al., 2005), and require interaction with a GTPase activating protein (GAP) to efficiently hydrolyze GTP and terminate action of the GTPase (Tetlow et al., 2013; Randazzo and Kahn, 1994). In the case of Arfs, the myristyl group snaps into a hydrophobic pocket within the protein, and Arf-GDP leaves the membrane within milliseconds of GTP hydrolysis (Antonny et al., 2005). It has been proposed that GAPs sometimes function as effectors, so their function can potentially be more complex than simple downregulation of the GTPase [reviewed in (23)].

## Discovery of arfs

The first protein in the Arf family, Arf1, was discovered in 1984 (Kahn and Gilman, 1984) as a factor required for cholera toxin A chain to ADP-ribosylate the Gs alpha subunit, the stimulatory component regulating adenylate cyclase. It was later independently identified by different groups as a factor required for membrane localization of COPI (Helms and Rothman, 1992; Donaldson et al., 1991; Teal et al., 1994). Rothman and coworkers had previously found a GTP requirement in a cell-free assay for transport within the Golgi apparatus (Melancon et al., 1987). By electron microscopy they found that coated pits were first formed on Golgi membranes in a GTP-dependent manner, followed by budding of coated vesicles and subsequent uncoating of these vesicles, with the uncoating depending on the hydrolysis of GTP (Serafini et al., 1991). Independently, Klausner and coworkers found that the drug brefeldin A caused merger of the Golgi and endoplasmic reticulum (Lippincott-Schwa et al., 1989) and that a very early event (30″ after brefeldin A addition) was the GTP-hydrolysis-dependent loss of a 110 kD protein from Golgi membranes (Donaldson et al., 1990). In both cases, it was shown that the protein Arf1 (initially known as Arf) was responsible for the GTP dependence, while the 110 kD protein was found to be a component of the COPI complex, which was the coat identified by the Rothman lab. (Helms and Rothman, 1992; Donaldson et al., 1991; Serafini et al., 1991; Palmer et al., 1993).

Based on these results, it was proposed that Arf1 complexed with GTP recruited the coat protein COPI to Golgi membranes (Serafini et al., 1991). COPI would then be released when Arf1 hydrolyzed GTP. This cycle could be coupled to vesicle formation and uncoating or occur independent of vesicle formation (Presley et al., 2002). A similar cycle was shown to underlie the recruitment of clathrin adaptors, including AP’s (Stamnes et al., 1993; Traub et al., 1993) and GGA’s (Boman et al., 2000; Puertollano et al., 2001), to Golgi membranes.

## Arf family overview

The Arf family of GTPases is part of the Ras superfamily of small GTPases and is defined by homology to Arf1 (Kahn et al., 2006; Price et al., 1996). Subsequent to the discovery of Arf1, a series of highly related proteins were discovered. These proteins, the core Arfs in mammals are Arf1, Arf3, Arf4, Arf5 and Arf6 with some mammals (but not primates) also possessing Arf2 (Price et al., 1996). They can be grouped by homology into three classes which have nearly identical Switch 1 and Switch 2 sequences but differ in regions near their N- and C-terminal portions (Tsuchiya et al., 1991). The Class I Arfs are Arf1, Arf2 and Arf3 (Tsuchiya et al., 1991). Class II includes Arf4 and Arf5 (Tsuchiya et al., 1991). Arf6 is the only Class III Arf (Tsuchiya et al., 1991) but is not found on the Golgi apparatus, and instead plays a role in regulating endocytosis and actin dynamics (D’Souza-Schorey et al., 1995; Peters et al., 1995). It is therefore beyond the scope of this review. In contrast, all Class I and Class II Arfs can be found on the Golgi apparatus (Tsai et al., 1992; Chun et al., 2008). The extent to which they have redundant versus distinct functions is not fully understood. However, given that amino acid sequences of the different Arfs are highly conserved between major mammalian groups such as rodents, ungulates, and humans (Price et al., 1996) there appears to be evolutionary pressure keeping them separate (Price et al., 1996). Based on phylogenetic evidence it has been proposed that Class I and Class III Arfs existed in the Last Eukaryotic Common Ancestor (LECA) with Class II Arfs (found in vertebrates, *Caenorhabditis elegans*, and *Drosophila melanogaster* but not in *Saccharomyces cervesiae*) diverging from Class I Arfs prior to the evolution of metazoans (Vargova et al., 2021).

Additionally, the family contains a number of other Arf-related proteins with lower homology for a total of 21 members in humans, including Sar1a and b, various Arf-related proteins (Arls) and Trim23 (Kahn et al., 2006; Kahn et al., 2005). These non-core Arf-related proteins are reviewed elsewhere [e.g. (Sztul et al., 2019; Kahn et al., 2005)].

**FIGURE 1:**
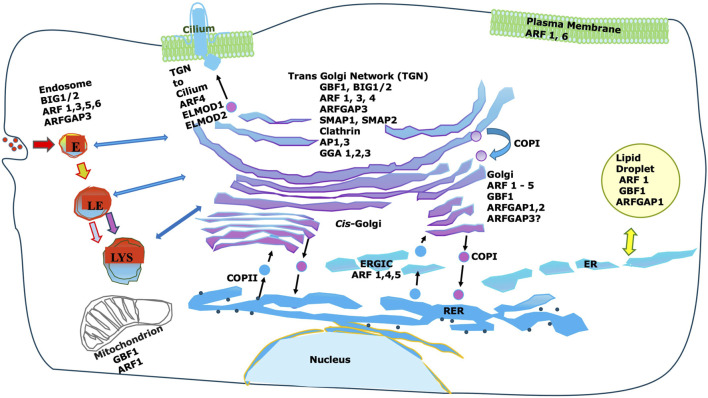
Intracellular localization of Arfs and important Arf interactors. While Arfs are shown there are many non-Golgi GEFs, GAPs and effectors not shown. Furthermore, most non-coat effectors are not shown. Note that Arf effectors can vary dramatically between different membranes, and that Golgi-localized GEFs and GAPs are frequently also localized to non-Golgi membranes.

Remarkably, while there are only five core Arf proteins in humans (six in many other mammals), they are regulated by numerous GEFs and GAPs [reviewed in (Sztul et al., 2019)]. These regulatory proteins show considerable membrane specificity, suggesting that, as has been seen in Rho family GTPases (Duman et al., 2015), the specificity of action of Arfs may be determined in part by their regulatory proteins (see Figure 1).

## Class I arfs

The initial research focused on Class I Arf, Arf1, specifically on its recruitment of the COPI coat. However, it was subsequently found to recruit some clathrin coats as well. This fact, that Arf1 plays a similar role in recruiting the clathrin adaptor proteins AP-1 and GGA to *trans*-Golgi-network (TGN) membranes (Stamnes et al., 1993; Puertollano et al., 2001) as it does in recruiting COPI suggested an unexpectedly complex regulation considering that distinct coats were recruited to different membranes by the same GTPase. It was further found that some of these TGN-localized clathrin adaptors are also found on endosomes [reviewed in (Sztul et al., 2019; Inoue and Randazzo, 2007)].

This simple picture of Arf1 as primarily regulating membrane association of COPI was upended by the discovery that Arf1 also activated phospholipase D on Golgi membrane (Ktistakis et al., 1995). While PLD activation would produce phosphatidic acid which would favor negative curvature in membranes and thus facilitate vesicle budding, it was not itself a part of the COPI coat machinery. Further roles for Arf1 on the Golgi apparatus were reported including recruitment of spectrin (Godi et al., 1998), actin (Fucini et al., 2000), and a PI4K complex (Godi et al., 1999; Highland and Fromme, 2021). This suggested that Arf1 interacted with a multitude of effectors and had functions additional to direct involvement in coat recruitment.

Subsequently, Arf1 was shown to be present on other membranes, including endosomes (Faundez et al., 1998), the plasma membrane (Norman et al., 1998), mitochondria (Ackema et al., 2014), and even lipid droplets (Nakamura et al., 2004) (Figure 1). Some of these functions could be linked to coat recruitment as the adaptor proteins GGA and AP-3, both recruited by Arf1, can be found on endosomes in addition to the TGN (Faundez et al., 1998). However, it is clear that Arf1 recruits many effectors including multiple coats and also non-coat effectors (Figure 1).

Overall, as more functions were discovered, it became clear that Arf1 regulates multiple functions on a variety of cellular membranes, with many of these functions being specific to particular membranes. This mirrors the complexity and context dependence seen in other Ras-family proteins, in particular the Rho family, e.g., Rac/Rho/Cdc42, where a small number of GTPases can have a great variety of potential functions depending on their sites of action [reviewed in (Duman et al., 2015)].

Most studies of Class I Arfs have concentrated on Arf1, however most mammals also possess Arf2 and Arf3. Arf2 was lost in primates and its role is poorly understood (Price et al., 1996). However, HA-tagged murine Arf2 was shown to localize robustly to the Golgi in Vero cells (Hosaka et al., 1996). Similarly, we found that GFP-tagged bovine Arf2 localized to the Golgi apparatus in Vero and NRK cells (Dejgaard et al., 2008). This would suggest one or more roles for Arf2 on the Golgi apparatus which may have been redistributed to Arf1, Arf3, Arf4, or Arf5 during the evolution of humans. Unfortunately, very little work has been done on Arf2, likely due to its absence in humans, and what its specific functions could be are not currently understood.

Arf3 is notable because it is well-conserved in mammals and differs from Arf1 by only seven amino acids, four near the N-terminus and three near the C-terminus (Tsuchiya et al., 1991). Despite this, it appears to localize specifically to TGN membranes with this TGN-specific localization dependent on C-terminal residues A174 and K178, which differ between Arf3 and Arf1 (Manolea et al., 2010). In that study, siRNA knockdown of the Arf GEFs BIG1 and BIG2 abrogated Golgi localization of Arf3, and TGN localization of Arf3 could be protected from brefeldin A treatment by overexpression of BIG1, but not the Arf GEF GBF1. Another group reported that BIG1 triggers a PI(4,5)P2-mediated macrophage-proinflammatory response via Arf3, and that Arf3 but not Arf1 physically interacted with BIG1 in a pulldown assay (Liu et al., 2020).

Additional evidence for specialization of function is suggested by the fact that dominant mutations in Arf3 have been found to cause developmental disorders in humans (Fasano et al., 2022; Sakamoto et al., 2022) that could be recapitulated in zebrafish (Fasano et al., 2022). In another study, Arf3 was shown to regulate turnover of N-cadherin in prostate cancer cells (Sandilands et al., 2023), although their evidence suggested an effect related to recycling endosomes rather than the secretory pathway. Co-depletion of both Class I Arfs (Arf1 and Arf3) triggers tubulation of recycling endosomes and retards recycling of transferrin receptor (Tf-R), suggesting Class I Arfs play a role on this organelle (Kondo et al., 2012). In this study, the phenotype could be rescued by overexpression of either Arf, indicating the function was redundant (Kondo et al., 2012).

## Class II arfs

In humans, Arf4 and Arf5 are the Class II Arfs. There is evidence that Arf4 is found on pre-Golgi vesicular-tubular complex (VTC) membranes and early Golgi compartments (Chun et al., 2008) within the secretory pathway. Arf5 appears to have a similar distribution on Golgi but is less prominent on VTCs (Chun et al., 2008). Like the Class I Arfs, both Arf4 and Arf5 can support COPI vesicle budding *in vitro* (Popoff et al., 2011), suggesting some redundancy.

Fairly extensive studies have reported that Arf4 is required for transport from TGN to cilia (reviewed in Deretic et al., 2021), although it has been argued to be non-essential for this transport pathway (Follit et al., 2014). The Class II Arfs have also been identified as required for replication of some viruses (Kudelko et al., 2011; Iglesias et al., 2015; Farhat et al., 2016; Ferlin et al., 2018; Neufeld et al., 2019). Both Arf4 and Arf5 have multiple roles outside of the Golgi apparatus, with depletion of Arf4 together with Arf1 resulting in tubulation of recycling endosomes and impaired recycling of mannose-6-phosphate receptor from endosomes to the TGN, but without affecting internalization or recycling of the transferrin receptor (Nakai et al., 2013; Volpicelli-Daley et al., 2005). Arf4 is also involved in a Golgi stress pathway, suggesting that its role is not limited to membrane trafficking (Reiling et al., 2013). Interestingly, mammalian cells can survive with Arf4 alone if Arf1, Arf3, and Arf5 are deleted (Pennauer et al., 2021), however deletion of Arf4 alone was not found to be lethal in the tissue culture cells tested in this study (Pennauer et al., 2021), although Arf4 deletion is embryonic lethal in mice (Follit et al., 2014).

Overall, these findings pose important questions which will require specific determination of mechanisms of action of Class II Arfs, particularly Arf4, to answer.

## Evidence from knockdown and imaging experiments

Kahn and coworkers originally attempted to address the need for multiple Arfs with siRNA knockdowns of all Class I and Class II Arfs in HeLa cells (Volpicelli-Daley et al., 2005), examining the morphology of intracellular organelles and coat proteins as well as utilizing functional assays for the vesicular stomatitis virus glycoprotein tagged with GFP (VSVG-GFP) trafficking to the cell surface, the lysine aspartic acid glutamic acid leucine receptor (KDELR) trafficking, and endosomal trafficking of the Tf-R. Knockdown of any single Arf gave no phenotype. However, knockdown of any pair of Arfs gave a phenotype in at least some assays. Double knockdown of Arf1+Arf3 or of Arf1+Arf4 altered the distribution of COPI and the localization of KDELR while also impairing the trafficking of VSVG-GFP to the cell surface. Arf1+Arf5 or Arf3+Arf4 knockdown altered the distribution of KDELR, suggesting cycling between ER and Golgi had been affected. The other combinations tested (Arf3+Arf4; Arf3+Arf5) impaired recycling of the transferrin receptor back to the cell surface but didn’t show Golgi phenotypes in the assays tested. Notably, all other combinations except Arf1+Arf4 showed effects on transferrin receptor recycling as well (Volpicelli-Daley et al., 2005). This study was consistent with a distribution of distinct functions between different Arfs, but also a considerable degree of redundancy.

A subsequent study (Pennauer et al., 2021) used CRISPR/Cas9 to delete the different Golgi Arf genes, also in HeLa cells. In contrast to the previous study, they found an enlarged Golgi with less βCOP, AP1γ1 and GGA2 per unit of Golgi area when Arf1 alone was deleted, as well as slight growth defects when either Arf1 or Arf4 were deleted. Notably, the effect of Arf1 knockout on coat recruitment could not be rescued by overexpression of Arf3, which differs from Arf1 by only seven amino acids.

**FIGURE 2:**
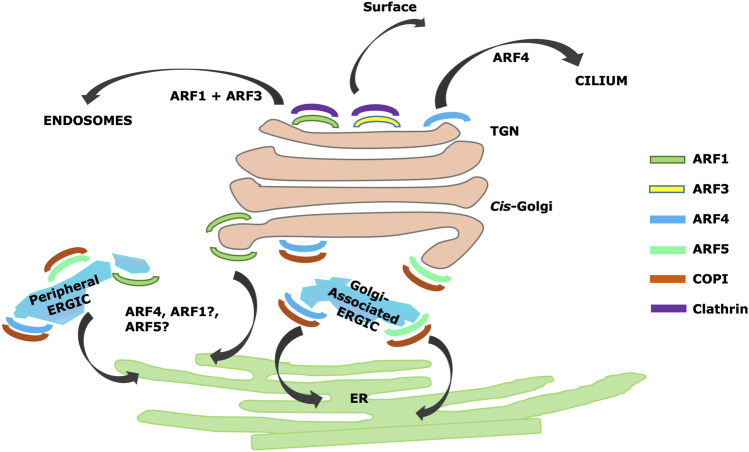
Summary of Arf localizations and activities identified by CRISPR/Cas9 knockdown (Pennauer et al., 2021) and superresolution microscopy (Wong-Dillworth et al., 2023), including proposed TGN localization and roles for Arf4 in trafficking to cilia (Deretic et al., 2021).

Knockout of Arf4 alone or together with Arf5 resulted in ER resident proteins including BiP, calnexin, and calreticulin reaching the cell surface or escaping the cell and also resulted in accumulation of KDEL receptor in the Golgi apparatus, suggesting a strong block in at least some retrograde trafficking pathways (Pennauer et al., 2021) (Figure 2). In contrast, knockdown of Arf4 alone by siRNA (Volpicelli-Daley et al., 2005) had no effect on measured levels of COPI on Golgi. The discrepancy between the two studies could be due in part to the fact that the gene was removed by CRISPR/Cas9 by Pennauer and coworkers (Pennauer et al., 2021), while the earlier study (Volpicelli-Daley et al., 2005) utilized siRNA knockdown, which can leave some residual expression. As Pennauer analyzed only a single clone for each CRISPR/Cas9 knockout, clonal variation unrelated to the Arf deletions in this study can also not be ruled out, although they did show that the major phenotypes identified could be rescued by lentiviral expression of the deleted Arf(s).

In the study by Pennauer (Pennauer et al., 2021), knockout of Arf4 or Arf5 also had no effect on recruitment of TGN-localized clathrin adaptors, suggesting a major role for Class I Arfs in sorting at the TGN. Surprisingly, Pennauer and coworkers reported that viability required only Arf4, as a triple knockout of (Arf1+Arf3+Arf5) survived (Pennauer et al., 2021). There was not an analysis of clathrin-dependent sorting from the TGN in cells lacking Class I Arfs or even the Arf1+Arf3+Arf5 knockout. However, these knockout combinations are viable, suggesting some residual activity in all essential pathways and implying that Arf4 can recruit all essential coats, although not necessarily at wild-type levels.

Wong-Dilworth and coworkers (Wong-Dillworth et al., 2023) further systematically investigated the intra-Golgi localization of different Arfs using STED microscopy, which is a super-resolution confocal microscopy-based technique. In this study, they used CRISPR/Cas9 knock-in to introduce HaloTag and epitope-tagged Arfs into cells to examine their locations within the Golgi apparatus using superresolution microscopy, comparing their locations to several Golgins and the COPI and clathrin coats. The Golgins GM130, Golgin97, and GRASP65 were used as markers for the flat regions of Golgi cisternae, while the coats were used as markers for the curved rims of cisternae. ERGIC53 was used as a marker for endoplasmic reticulum–Golgi intermediate compartment (ERGIC) (Wong-Dillworth et al., 2023) a fraction of which was reported to be directly apposed to *cis* Golgi membranes (Wong-Dillworth et al., 2023). While the markers chosen for various structures were plausible, a caveat is that there was no morphological confirmation of the nature of the structures by electron microscopy.

In this study, Arf1 was reported to be on *cis*-Golgi cisternae, but excluded from *cis*-Golgi apposed ERGIC, and also on TGN membranes (Wong-Dillworth et al., 2023). Arf4 and Arf5 were reported to be on both peripheral and Golgi-apposed ERGIC (Wong-Dillworth et al., 2023). Interestingly, Arf4 and Arf5 appeared to be localized to domains on ERGIC (Wong-Dillworth et al., 2023) consistent with previous reports (Chun et al., 2008) but clearly segregated from each other in the super-resolution images. Small amounts of Arf1 were found on peripheral ERGIC, but largely not overlapping with Arf4 (Wong-Dillworth et al., 2023). The authors suggested that different Arfs localize to different microdomains with specialized function (Figure 2). In a previous study, the same laboratory had imaged Arf1 leaving the Golgi in thin tubules, some of which had attached COPI and which may have been involved in Golgi to ER retrograde trafficking (Bottanell et al., 2017). They therefore proposed that Arf1 is involved in retrograde traffic.

They found Arf4 on regions of apparent ERGIC not containing Arf1 and suggested Arf4 was involved in anterograde traffic. However, given the clear effects of Arf4 knockdown inhibiting retrograde trafficking of KDEL receptor in the two previously discussed studies (Volpicelli-Daley et al., 2005; Pennauer et al., 2021), it is likely Arf4 is involved in retrograde traffic in some manner. One explanation could be that Arf4 or Arf1 are present at different stages on or during the formation of the same retrograde structure. Another is if Arf4 and Arf1 are both involved in retrograde trafficking but in distinct pathways. Notably, there is evidence for at least two distinct retrograde pathways, one dependent on COPI and generally considered to involve COPI coated vesicles (Letourneur et al., 1994) and a second pathway involving tubules dependent on Rab6 and independent of COPI (White et al., 1999). Although the Rab6 pathway originates on TGN membranes and is likely not involved in retrograde trafficking of KDEL receptor, additional pathways may exist. Regardless, the work by Wong-Dillworth and coworkers (Wong-Dillworth et al., 2023) suggests a surprising degree of specialization of Arfs 1, 4 and 5 on ERGIC and early Golgi membranes.

Similarly, Wong-Dillworth and coworkers reported that only Arf1 and Arf3 were present on TGN membranes, with both colocalized on the same clathrin-positive tubules (Wong-Dillworth et al., 2023). Arf3 could be found on late Golgi cisternae but was interestingly concentrated in small domains from which Arf1 was excluded (Wong-Dillworth et al., 2023). This is consistent with specialized roles for Arfs 1 and 3. The TGN is involved in the formation of a variety of different transport intermediates, both clathrin and non-clathrin, targeted to different domains on the cell surface as well as to the endosomal pathway (Ramazanov et al., 2021). The TGN (Griffiths and Simons, 1986) also possesses other roles, including proteolytic modifications of proteins (Xu and Shields, 1993; Robakis et al., 1993), receiving transport intermediates from endosomes by the retromer and other pathways (Buser and Spang, 2023), and production of sphingolipids and glycosphingolipids (Kobayashi et al., 1992).

## Arf GEFs, arf receptors and mechanisms of arf localization and specialization

There are at least 15 Arf GEFs in the human genome organized into six families (reviewed in (Sztul et al., 2019)). Of these, only one (GBF1) is found on the Golgi proper (Zhao et al., 2006). GBF1 is also found on the TGN (Lefrancois and McCormick, 2007), lipid droplets (Ellong et al., 2011), and the cell surface (Busby et al., 2017). ARFGEF1/BIG1 and ARFGEF2/BIG2 are also found on TGN membranes (Zhao et al., 2002) in addition to some endosomes (reviewed in Sztul et al., 2019). Other Arf GEFs are found on various non-Golgi membranes, including endosomal membranes, plasma membrane, postsynaptic densities, and tight junctions (reviewed in Sztul et al., 2019). There is evidence that some of these GEFs also recruit Arf effectors, which could possibly be handed off to newly-recruited Arfs (Lefrancois and McCormick, 2007; Deng et al., 2009; Manolea et al., 2008).

As discussed previously, there are differences in the localization of Golgi Arfs despite substantial interchangeability in function. This is in part affected by the localization and specificity of Arf GEFs. There is evidence that Arf3 is recruited by BIG1/BIG2 (Manolea et al., 2010; Liu et al., 2020), found on TGN membranes, while GBF1 recruits multiple other Golgi Arfs to pre-Golgi and early Golgi membranes (Chun et al., 2008; Claude et al., 1999), although GBF1 is also present on TGN membranes (Lefrancois and McCormick, 2007; Lowery et al., 2013; Wang et al., 2017). Evidence suggests that activated Rab1b brings GBF1 to pre-Golgi and early Golgi membranes (Alvarez et al., 2003; Monetta et al., 2007), possibly with a role for Rab1b effectors such as PI4KIIIα (Dumaresq-Doiron et al., 2010). Consistent with this, Meissner and coworkers have provided direct evidence that GBF1 interacts with membranes via a phosphoinositide-binding HDS1 domain, and that it has a requirement for PI(3)P, PI(4)P or PI(4,5)P_2_ (Meissner et al., 2018). How GBF1 is recruited to TGN membranes is unclear, but a role there also for phosphoinositide binding would be plausible. Some TGN-localized clathrin adaptors are also recruited by phosphoinositides (Crottet et al., 2002; Gaidarov et al., 2005; Wang et al., 2003; Wang et al., 2007; Daboussi et al., 2012). This highlights that, while Arf GEFs and Arfs form part of a pathway for the recruitment of effectors, there are other upstream elements that could also play a role.

Some studies had provided evidence that double knockdown of both BIGs leads to failure to recruit GGA1-3 adaptors to the Golgi apparatus (Manolea et al., 2008; Ishizaki et al., 2008). However, this was contradicted by evidence that GGA recruitment to TGN depended on GBF1 activity and, interestingly, also that GBF1 bound directly to the VHS-GAT domain of GGAs (Lefrancois and McCormick, 2007). A subsequent study provided insight into the previous contradictory reports, suggesting a more complex and interdependent regulation than previously suspected. According to Lowery and coworkers GBF1 on TGN membranes recruits Class II Arfs (i.e., Arf4 and Arf5), and the Class II Arfs then recruit BIG1 and BIG2 (Lowery et al., 2013). TGN-localized GBF1 complexed with rhodopsin, a light-sensitive protein in rod photoreceptor cells that depends on cilia trafficking for its essential function for vision, has also been proposed to recruit Arf4 in support of ciliary trafficking. However, this pathway does not necessarily originate from the same TGN nanodomains as the more conventional clathrin vesicles.

As BIG1/2 appears to preferentially recruit Arf3 (Manolea et al., 2010; Liu et al., 2020), this sequence of events is somewhat reminiscent of Rab cascades, e.g., Rab5 on early endosomes recruiting exchange factors for the late endosomal Rab7 [reviewed in (Borchers et al., 2021)]. Rab7 in turn inhibits recruitment of Rab5, driving the irreversible transformation of an early endosome to a late endosome (Borchers et al., 2021). In contrast to endosomes, the TGN consists of a poorly understood mosaic of domains which will produce a variety of distinct transport intermediates including both clathrin coated and uncoated intermediates (reviewed in Ramazanov et al., 2021). Therefore, the ecology of GEF-Arf interactions could be a less linear and more complex process. This will require further research.

Besides GEFs, another important factor regulating Arf localization may be the recruitment of Arfs by membrane receptors upstream of GEFs. In particular, there is evidence that Arf recruitment to Golgi membranes is facilitated by non-effector Arf receptors (i.e., capable of binding to Arf-GDP). The ER-Golgi SNARE membrin was shown to bind to Arf1-GDP and to be required to recruit Arf1 to early Golgi but not to TGN membranes (Honda et al., 2005). This targeting depended on the motif MXXE which is found in all Golgi-localized Arfs, but which is lacking from the non-Golgi-localized Arf6 (Honda et al., 2005). A second protein, the COPI-accessory protein Scyl1, was shown to recruit Arf4-GDP to Golgi membranes and ERGIC (Hamlin et al., 2014). While Scyl1 preferentially interacted with Arf4, it showed some binding to Arf5 as well, but not to Arf1 or Arf3, and was proposed to link class II Arfs to γ2-bearing COPI subcomplexes (Hamlin et al., 2014). This binding disappeared after mutation of residues in Arf4 opposite the nucleotide-binding pocket (Hamlin et al., 2014). These interactions could serve to locally increase the concentration of Arf-GDP, leading to more efficient action of the exchange factor.

There could be multiple unknown receptors, as neither Scyl1 nor membrin are found on TGN or endosomal membranes. Identifying receptors on different membranes will be critically important in understanding the mechanisms of Arf targeting, as different receptors may have different specificities (e.g., membrin seems to recruit all Golgi Arfs (Honda et al., 2005), while Scyl1 is specific for Class II Arfs [Hamlin et al., 2014)]. As a general feature of membrane receptors would be the ability to efficiently bind to Arf-GDPs, it might be possible to identify them using BioID or similarly-tagged GDP-locked Arf mutants. However, recent studies of Arf interaction partners using biotin proximity tagging have used wild-type or GTP-locked Arf mutants, which would not distinguish GTP-independent binding from effector binding (Quirion et al., 2024; Li et al., 2022).

## Arf GAPs on the Golgi apparatus

A mammalian Arf GAP (ArfGAP1) was originally isolated from rat liver cytosol and shown to be localized to the Golgi apparatus (Cukierman et al., 1995). This was followed by the discovery in yeast of two Arf GAPs. One, Gcs1p (Poon et al., 1996), was highly homologous to ArfGAP1, but appeared to be partially redundant with the less homologous Glo3p (Poon et al., 1999; Dogic et al., 1999).

As Arf1 was known to recruit COPI when activated and to release it subsequent to vesicle uncoating, ArfGAP1 was regarded as an essential component of this cycle with the role of triggering GTPase activity in Arf1 to facilitate vesicle uncoating. Work by Goldberg suggested that COPI directly activated ArfGAP1 *in vitro* (Goldberg, 1999). Randazzo and coworkers also found evidence for direct activation by COPI (Luo and Randazzo, 2008), while Antonny and coworkers provided evidence that ArfGAP1 activity required membrane curvature similar to that expected in a COPI vesicle (Bigay et al., 2003) and that ArfGAP1 sensed curvature through a lipid-packing sensor (Bigay et al., 2005), failing to bind to flat membranes, but binding efficiently to tubules with a radius of approximately 35 nm that had been pulled out of the same flat membranes using molecular motors or optical tweezers (Ambroggio et al., 2010). Based on this, they concluded that ArfGAP1 could bind to curved membranes in COPI buds. In their model, ArfGAP1 could cause loss of Arf1 from COPI buds, with this Arf1 rapidly replenished by diffusion from neighboring membranes prior to budding (Ambroggio et al., 2010). ArfGAP1 activity would continue subsequent to vesicle budding, but as Arf1 would now not be replenished, the vesicle would uncoat. While these data supported a simple model for ArfGAP1 action, uncoated tubules containing highly curved membranes are abundant next to Golgi membranes connecting adjacent stacks (Rambourg and Clermont, 1986), as well as playing roles in retrograde (White et al., 1999) and forward (Hirschberg et al., 1998; Toomre et al., 1999) trafficking out of the Golgi, suggesting ArfGAP1 could be active away from COPI buds. Additional studies suggested that ArfGAP1 aids the assembly of COPI on Golgi membranes (Yang et al., 2002; Shiba et al., 2011). Although this was contested (Beck et al., 2009), this raises the possibility that ArfGAP1 acts as an Arf effector and that its role could be more complex than previously appreciated (Kahn, 2011).

Subsequently, two Glo3p homologous proteins (ArfGAP2 and ArfGAP3) were identified in mammals (Frigerio et al., 2007), and they were reported to be on pre-Golgi and early Golgi membranes together with ArfGAP1 (Frigerio et al., 2007). In this study, ArfGAP2 and ArfGAP3 were found to colocalize with COPI by immunofluorescence in normal rat kidney (NRK) cells, and to be present in COPI vesicles generated *in vitro* in the presence of GTPγS (Frigerio et al., 2007). They were also shown to bind directly to COPI by multiple groups (Weimer et al., 2008; Luo et al., 2009; Shiba et al., 2013). Arf1 was shown to interact with ArfGAP1 (Goldberg, 1999) and ArfGAPs 2 and 3 (Frigerio et al., 2007) on *cis*/*medial* Golgi membranes. Knockdown of either ArfGAP1 alone or ArfGAP2 and 3 together had no effect on cell viability, while a knockdown of all three Arf GAPs was lethal, consistent with some redundancy in function (Frigerio et al., 2007). Interestingly, retrograde trafficking of cholera toxin to the ER was inhibited when ArfGAP2 and 3 were knocked down (Frigerio et al., 2007). Further studies confirmed that ArfGAPs 2 and 3 bound specifically to COPI (Weimer et al., 2008). Taken together, these findings suggest an important role for ArfGAPs 2 and 3 on COPI vesicles and for retrograde trafficking. However, the division of labor between GCS1 homologue ArfGAP1 and the two GLO3 homologues (ArfGAP2, ArfGAP3) is still not clear.

Other later work suggests that while ArfGAP3 can interact with COPI (Kliouchnikov et al., 2009), it is primarily found on the TGN and endosomes, is required for proper trafficking of the mannose-6-phosphate receptor, and in addition to COPI can bind directly to the clathrin adaptor GGA (Shiba et al., 2013). This suggests a degree of specialization with ArfGAP2 playing a greater role on COPI-containing early Golgi membranes and ArfGAP3 preferentially interacting with elements of the clathrin machinery. Interestingly, knockdown of ArfGAP3 reduced association of GGA1 with TGN in a light microscopic assay, consistent with ArfGAP3 playing a positive role in clathrin adaptor recruitment (Shiba et al., 2013), suggesting that, as may be the case with ArfGAPs 1 and 2, it is an Arf effector with GAP activity and not simply a negative regulator of Arf activity.

A total of twenty-seven proteins are currently identified as Arf GAPs (reviewed in Sztul et al., 2019). Of these, only three (ArfGAP1, ArfGAP2, ArfGAP3) have been reported on Golgi membranes proper, while a larger number have been reported on TGN membranes including ArfGAP3 (Shiba et al., 2013), SMAP1 (Kobayashi et al., 2014; Wang et al., 2021) and SMAP2 (Natsume et al., 2006; Matsudaira et al., 2013) with the TGN-localized GAPs also generally found on endosomes. ELMOD1 and 3 may be involved in Golgi to cilium trafficking, presumably from late Golgi compartments (Turn et al., 2022), and there is evidence that the Arf GAP ASAP1 is involved in TGN to cilia trafficking, together with Arf4 (Mazelova et al., 2009). Numerous other Arf GAPs have no known Golgi involvement but are found on various endosomal populations and on plasma membranes or subdomains thereof (reviewed in Sztul et al., 2019).

The specificity of Arf GAPs for Arfs other than Arf1 is poorly understood. One recent study used biotin proximity ligation to identify the set of interactors of all Arf/Arl family proteins (Quirion et al., 2024). In this study, ArfGAP1, ArfGAP2, ArfGAP3, and SMAP2 could be tagged with biotin by any of Arf1, 3, 4, and 5, suggesting interaction. Interestingly, ArfGAP1 also showed proximity to some non-Arf Arl proteins (Quirion et al., 2024). The Class I Arfs (Arfs 1 and 3) were also seen to interact with AGFG1 (also known as RIP1), an Arf GAP involved in regulating cell death. While ELMOD1, 2 and 3, and ASAP1 were not identified as Arf interactors in this study, it is possible that the pathways they regulate (e.g., trafficking to cilia) are not sufficiently prominent in the cell lines used (HeLa and HEK293).

## Arf effectors

The best-studied Arf effectors are the COPI and other coats. While the interaction of Arfs with COPI is complex, recent structural studies of COPI coats assembled on membranes *in vitro* and *in vivo* have created considerable insight into its interaction with Arf1. COPI subunits are stable structures composed of seven polypeptides (α, β, β′, γ, δ, ε and ζ). These subunits are divided into two major subcomplexes. One subcomplex consists of α, β′ and ε subunits and is considered to structurally correspond to clathrin or to the outer (Sec13/31) subunits of the COPII coat. The other subcomplex consists of β, γ, δ and ζ and can be divided into two homologous pairs (β/δ and γ/ζ) possibly created by an ancient gene duplication event and homologous to the major subunits of the AP family of clathrin adaptors. Early studies using chemical cross-linking and yeast two-hybrid suggested multiple binding partners for Arf1. Yeast two-hybrid analysis suggested an interaction of Arf1 with α-COP and β-COP (Eugster et al., 2000). Initial cross-linking experiments confirmed an Arf1 interaction with β-COP (Zhao et al., 1997) but also with γ -COP (Zhao et al., 1999). Arf1 could be photo-crosslinked simultaneously to β-COP and δ-COP (Sun et al., 2007), and in the same study was found also to bind to γ -COP and to β′-COP (Sun et al., 2007). Sun and coworkers proposed that a single coatomer complex possessed more than one binding site for Arf1.

A subsequent study (Yu et al., 2012) provided structural and biochemical evidence that the β/δ and γ/ζ subcomplexes could each bind Arf1 independently. Yu and coworkers further purified crystals of the γ/ζ complex bound to Arf1 and determined the structure to 2.9 Å resolution. Further cryo-electron microscopic structural work (Dodonova et al., 2017) of *in vitro* assembled COPI coats confirmed that two Arf1 molecules per coatomer complex were found in the COPI coat, one associated with the β/δ subcomplex and the other with the γ/ζ subcomplex. δ-COP was found to interact directly with the Arf1 Switch 1 region consistent with nucleotide-dependent binding. This group proposed that the GTP-dependent interaction with δ-COP is important for recruitment of COPI to membranes by Arf1 and also suggested that Arf1 could potentially modulate the conformation of δ-COP. A second Arf1 was identified interacting with γ/ζ, with direct binding only to γ-COP. In their structure this γ-COP-bound Arf1 and its environment (neighboring β and β′COP subunits) forms a niche into which ArfGAP2 can bind. This bound ArfGAP2’s catalytic domain is positioned near the Arf1 nucleotide-binding site optimally for stimulation of GTP-hydrolysis. In their model the β/δ -subcomplex-bound Arf1 appears to be in a different molecular environment not accessible to ArfGAP2. They speculate that ArfGAP1 may regulate GTP hydrolysis of β/δ -subcomplex-bound Arf1, however knockdown of ArfGAP1 was not found to be lethal (Frigerio et al., 2007).

Arfs on TGN and endosomes were also shown to recruit clathrin adaptors AP-1, AP-3 and AP-4, which are multi-subunit complexes with homology with the β/δ and γ/ζ subcomplexes of COPI, and the GGA family (GGA1, GGA2 and GGA3) which consist of a single polypeptide chain. Both families of adaptors act to bind sorting signals on the cytoplasmic domains of cargo proteins and recruit them to nascent coated pits while also recruiting an outer clathrin coat, which drives membrane curvature and favors vesicle budding.

Early crosslinking experiments, as for COPI, suggested interaction of Arfs with multiple subunits of AP-1 (Austin et al., 2002). In this study Arf1, Arf5 and Arf6 were tested and found to crosslink to the β1 subunit (homologous to β-COP) but only Arf1 and Arf6 to the γ subunit of AP-1 (homologous to γ-COP) *in vitro* in the presence of GTPγS. Subsequently, the core part of the AP-1 complex was co-crystallized with ARF1-GTP (Ren et al., 2013), confirming that two molecules of Arf1 were bound to this core, one to the β1 subunit, and one to the γ subunit. Mutation of either binding site abrogated localization of the AP-1 core to TGN membranes indicating both were essential (Ren et al., 2013). They further provided evidence for a synergistic effect of cargo, Arf1, and PI(4)P on AP-1 binding to TGN (Ren et al., 2013).

A similar study (Begley et al., 2024) used cryo-EM of AP-3-Arf1 complexes assembled onto nanodisks to determine the structure. As with AP-1 two Arf1 binding sites were identified, one to the δ subunit (homologous to the γ subunit of AP-1) and one to the β3 subunit. In these structures either the δ subunit binding site alone was occupied or both. They proposed that the δ subunit was initially occupied followed by binding of a second Arf1 molecule to the β3 subunit with lateral polymerization of AP-3 occurring after both Arf1 molecules were bound (Begley et al., 2024).

The GGA adaptors (GGA1, GGA2 and GGA3) are much simpler in structure than the AP’s or COPI, being composed of a single peptide with a N-terminal cargo-binding VHS domain which recognizes a DXXLL sorting sequence (AL, 2001), an Arf-binding GAT domain (Collins et al., 2003; Suer et al., 2003) required for membrane recruitment followed by the hinge and GAE domain (AL, 2001). Both the hinge and GAE domain are required for optimal binding of clathrin. GGAs recruited *in vitro* to membranes by Arf1 can drive the polymerization of clathrin, largely into conventional basket-like coats but also into tubules (Zhang et al., 2007).

Arf1 may play a role in vesicle scission distinct from its role in coat recruitment. Beck and coworkers provided evidence that dimerization of Arf1 leads to membrane curvature and that this is required for scission of COPI vesicles as the Y35A mutation, which blocks dimerization also blocks vesicle budding *in vitro* while still permitting coat recruitment (Beck et al., 2008). Diestelkoetter-Bachert extended this work and reported that after vesicle scission dimeric Arf1 was found exclusively on donor membranes, suggesting this is a distinct pool from COPI-bound Arf1 (Diestelkoetter-Bachert et al., 2020). Whether dimeric Arf1 or other Arfs might play a role in the scission of Golgi-originated clathrin vesicles has not been addressed. However, scission of COPII vesicles by the related small GTPase Sar1 has been proposed to occur by a related mechanism (H et al., 2014).

Tubules containing fluorescently-tagged Arf1 have been visualized in living cells (Bottanell et al., 2017) and it is possible that the formation of these tubules is driven by the presence of dimeric Arf1. COPI was also visualized on these tubules but whether it consisted of conventional coated pits along the tubule (presumably containing monomeric Arf1) or a distinct organization was not addressed. In a different model system Arf1 was shown to engage in clathrin-independent recruitment of AP-1 to tubules in cells expressing the HIV Nef protein, and in this pathological condition the role of Nef was likely to sequester MHC-I into these tubules (Hooy et al., 2022). As neither AP-3 nor AP-4 require clathrin, and AP-3 is known to assemble into tubular structures (Bowman et al., 2021), this suggests that Arfs can recruit coats to tubules through mechanisms similar to those involved in their recruitment into the classical spherical vesicles.

The first non-coat effector proposed for Arf was phospholipase D (Ktistakis et al., 1995; Brown et al., 1993). This enzyme removes the head group from phosphatidylcholine to make phosphatidic acid, favoring negative membrane curvature. There is evidence that this interaction facilitates the formation of both COPI (Ktistakis et al., 1995) and TGN-originated (Chen et al., 1997) vesicles. There is also evidence that Arfs activate a PI4 kinase on TGN (Highland and Fromme, 2021). Arfs have also been reported to activate multiple lipid flippases and transfer proteins on TGN membranes, including FAPP1 and FAPP2 (Godi et al., 2004). FAPP1 and FAPP2 bind directly to Arf and to PI(4)P (Godi et al., 2004) and are involved in transferring glucosylceramides between ER and TGN membranes. Their activity is important for Golgi synthesis of sphingomyelin and glycosphingolipids, which are elements of liquid ordered membrane domains. The flippase ATP8A1, which primarily flips phospholipids into the inner leaflet, appears not to be directly recruited by Arfs, but stimulates activity of BIG1 and BIG2 and results in increased Arf activity and recruitment of Arf effectors GGA1 and AP1 (Pocognoni et al., 2024).

Arfs and Arf-related proteins are also involved in recruiting tethering/Golgi matrix molecules to Golgi membranes. A number of GRIP-domain containing proteins such as golgin-245 have been shown to interact with the Arf-like protein Arl1. The related Grip-related Arf-binding (GRAB) domains interact with Arfs. Golgi tether GMAP210, which may be involved in homotypic tethering of *cis*-Golgi elements, was reported to bind to Golgi membranes through an ALPS motif at its N-terminus and through an Arf1-binding GRAB domain at its C-terminus (Drin et al., 2008; Cardenas et al., 2009). Another tether, Golgin160 is recruited to Golgi membranes by Arf1, and in turn plays a role in recruiting dynein to maintain the Golgi’s typical localization near the microtubule organizing center (Yadav et al., 2012). Additionally, Arfs play a role in recruiting spectrins to Golgi membranes (Godi et al., 1998) and spectrins may play a role in recruiting an Arf-dependent pool of actin to Golgi membranes (Fucini et al., 2000). Specific roles of different Arfs in recruiting Golgi matrix tethers and cytoskeleton has not been extensively researched. However, the tethering/scaffolding protein Rab6-interacting golgin (GORAB), also known as SCY1-like 1-binding protein 1 (SCYL1-BP1), was found to interact specifically with Arf5 and is involved in Rab6 recruitment to the Golgi (Egerer et al., 2015). GORAB appears to function as an effector of both Arf5 and Rab6 through the same binding site (Egerer et al., 2015). As it can also bind to Class II Arf receptor Scyl1, this highlights the potential complexity of interactions that lead to proper localization of Arf effectors.

## Conclusion and future directions

**FIGURE 3:**
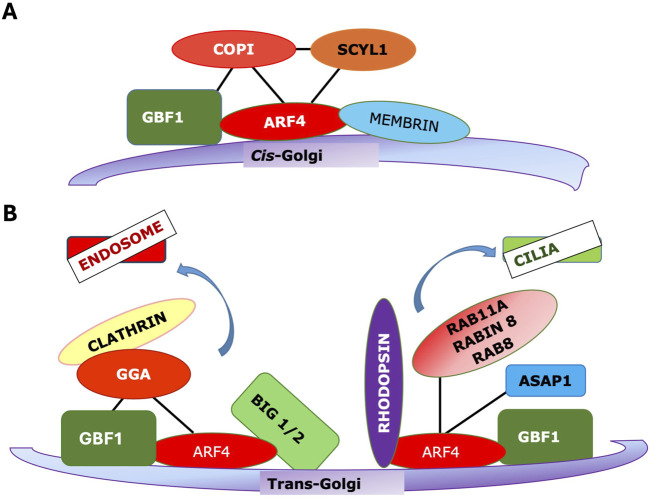
The same Arf/GEF combination can recruit distinct effectors to different membranes. **(A)** Arf4 can be recruited to cis-Golgi by GBF1, and in turn it recruits the COPI coat. **(B)** (left) Arf4 recruited to TGN membranes by GBF1 can recruit GGA and the Arf3 exchange factors BIG1/2 to support clathrin vesicle trafficking to endosomes (right) Arf4 can be recruited to TGN by GBF1 where, in distinct nanodomains, it binds rhodopsin and other factors to be trafficked to cilia.

An important feature of the action of Arfs, and one that will require a detailed mechanistic explanation, is the fact that the same Arf on different membranes recruits a distinct set of effectors. E.g., COPI is recruited exclusively to early Golgi membranes by Arfs (Figure 3A). Clathrin-adaptor proteins including the GGAs and AP-1 are present on TGN membranes (Figure 3B), while AP-3 and also GGAs are found on endosomal membranes. No clathrin adaptors bind to the early Golgi membranes to which COPI is recruited. This specificity is especially striking as Arf-regulated coats and many other effectors are recruited directly from a soluble cytoplasmic pool.

One possible explanation is that different Arfs recruit different coats along with distinct sets of other effectors. However, this cannot be a complete explanation, as any of Arfs 1, 3, 4 or 5 was able to support COPI vesicle formation in an *in vitro* assay (Popoff et al., 2011). In that report, Arf3 was excluded from the COPI vesicle fraction when any of Arfs 1, 4 or 5 were present, suggesting that Arfs may vary in efficiency for particular functions, but highlighting that the Arf is not the important determination of specificity here.

**FIGURE 4:**
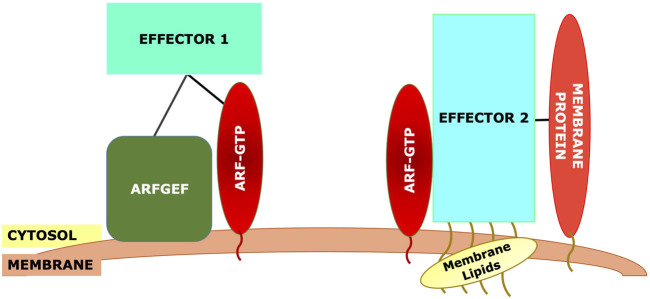
Generic model for recruitment of Arf effectors. The effector will typically require binding to the Arf-GTP. However, other interactions must be necessary to maintain specificity, as the same Arf may be present on other membranes. These other interactions could include: direct interaction with the Arf GEF, binding to membrane-specific Rab effectors (e.g., p115 in part A), binding to other membrane-specific proteins such as cargo proteins, or binding to modified membrane lipids such as phosphoinositides.

More likely, effector specificity depends on multiple interactions with the Arf interaction being essential, acting as a switch, but with other interactions also essential (Figure 4). This could include interactions with an Arf GEF, with Arf receptors, with other Arf effectors, with other resident proteins of the target membranes, either alone or in combination (Figure 4). There is evidence for all of these different interactions. GGA has been reported to bind directly to the Arf GEF GBF1 as assayed by pulldown (Lefrancois and McCormick, 2007). Similarly, GBF1 has been reported to bind directly to the γ subunit of COPI (Deng et al., 2009), while the Arf4 receptor Scyl1 (Hamlin et al., 2014) was originally identified as an interaction partner of COP1 (Burman et al., 2008). As GBF1 is found on TGN membranes as well, which lack COPI, additional determinants must exist. VTC and early Golgi membranes contain high concentrations of proteins such as the p24 family, which contain carboxy-terminal dilysine motifs (-KKXX) or other similar COPI binding motifs, which can increase the affinity of COPI for these membranes (Dominguez et al., 1998; Fiedler et al., 1996). Additionally, there is evidence that GBF1 on early Golgi membranes is recruited by Rab1b, which also helps to recruit COPI together with the tethering factor p115 (Guo and Linstedt, 2014) (Figure 3A).

In contrast, there is currently no clear evidence for involvement of Rab6 or other TGN Rabs in recruitment of GBF1 to TGN membranes. Here, GBF1 can bind directly to GGAs (Lefrancois and McCormick, 2007), as well as recruiting Arfs, including Class II Arfs, which further recruit the BIG1/BIG2 Arf exchange factors (Lowery et al., 2013). PI4KIIa localized on TGN, but not found on early Golgi compartments, is also required for the recruitment of at least AP-1 and GGA clathrin adaptors (Wang et al., 2007). A recent study (Pocognoni et al., 2024) provided evidence that the lipid flippase ATP8A1 binds directly to BIG1/BIG2 and that this flippase is involved in the recruitment of AP1, GGA2, and clathrin. This suggests that the recruitment of distinct effectors by Arfs depends in part on lipid composition of the target membrane and in part on the presence of other non-Arf proteins.

These studies taken together suggest that additional factors, and possibly lipid modifications, are required besides Arfs for the TGN-localization of Arf effectors. Some of these factors appear to be directly recruited by Arf GEFs, while others might be recruited to Golgi or TGN membranes by factors upstream of Arf GEFs or by independent mechanisms.

Some of these pathways, particularly those involving coat recruitment, are becoming better understood, and some basic principles can be distilled. First, the Arf is not the only determinant of specificity. Other factors, including GEFs, Arf receptors, membrane-localized proteins, and membrane lipids such as PIs, can also decisively influence effector recruitment. Second, while Arfs may have considerable functional overlap, the pathways they regulate on particular membranes are discrete and well-defined. Third, independent Arf-regulated pathways may exist on separate microdomains of a single membrane, with the best-characterized example being TGN membranes, where Arfs appear to play a role in forming various kinds of clathrin vesicles and as well to mediate trafficking to cilia via a clathrin-independent pathway (Figure 3B). In the TGN, it is likely that the pathways are spatially segregated on distinct nanodomains.

Many of the cited studies emphasize the linking of effector recruitment to early steps in Arf recruitment, with direct binding of a GEF to effectors identified in multiple studies. Additionally, at least one Arf receptor, the Arf4 receptor Scyl1, binds directly to the Arf effector COPI (Hamlin et al., 2014). Two recent studies (Quirion et al., 2024; Li et al., 2022) used proximity biotinylation as a high-throughput method to detect Arf interactors, but using wild-type or GTP-locked Arfs. Known effectors and GAPs were robustly identified, but no attempt was made to identify interactions that were not GTP-dependent, and the known GEFs were notably underrepresented in the detected proteins. While Arf GEFs are relatively well characterized, there may well be unknown Arf receptors, and proximity biotinylation using Arf-GDP as bait could be one possible approach for finding them. Additionally, given that early events in Arf recruitment may be critical in effector recruitment, identification of additional interaction partners of Arf GEFs and known Arf receptors may provide insight into the mechanisms by which Arf effector specificity is maintained. Notably, Arf GEFs in some cases are recruited by Rab proteins, raising the possibility that some of the specificity of effector recruitment may be upstream of the GEFs. Thus, Arf effector recruitment is not simply a consequence of the ability of the effector to bind Arf-GTP but needs to be understood as part of a larger pathway operating in the context of the local membrane environment.
